# Efficacy of Essential Oil of *Algrizea minor* Sobral, Faria & Proença (Myrtaceae) in Combating Methicillin‐resistant *Staphylococcus aureus*: Antibacterial and Virulence Modulation Studies Conducted In Vitro and In Vivo

**DOI:** 10.1002/cbdv.202501177

**Published:** 2025-07-10

**Authors:** Amanda Vieira de Barros, Bruno Oliveira de Veras, Henrique Nelson Pereira Costa Júnior, Raudiney Frankilin Vasconcelos Mendes, Patryck Érmerson Monteiro dos Santos, Jael Fernandes Tavares, Daniela Maria do Amaral Ferraz Navarro, Rafael Matos Ximenes, Patrícia Maria Guedes Paiva, Clovis Macedo Bezerra Filho, Josinaldo Alves da Silva, Márcia Vanusa da Silva, João Paulo Martins de Lima, Henrique Douglas Melo Coutinho, Maria Betânia Melo de Oliveira

**Affiliations:** ^1^ Department of Biochemistry Federal University of Pernambuco Recife Brazil; ^2^ Department of Antibiotics Federal University of Pernambuco Recife Brazil; ^3^ Department of Fundamental Chemistry Federal University of Pernambuco Recife Brazil; ^4^ School of Health and Life Sciences Catholic University of Pernambuco Recife Brazil; ^5^ Biosciences Center Federal University of Pernambuco Recife Brazil; ^6^ CECAPE College Juazeiro do Norte Brazil; ^7^ Department of Biological Chemistry Regional University of Cariri Crato Brazil

**Keywords:** amoxicillin, antibiotic resistance modulation, *Galleria mellonella*, natural compounds, terpenes

## Abstract

Infections caused by methicillin‐resistant *Staphylococcus aureus* (MRSA) are difficult to treat due to its resistance profile and the scarcity of new antibiotics, necessitating alternative therapeutic strategies. This study explores the antibacterial, antivirulence, and in vivo effects of *Algrizea minor* essential oil (EOAM) against MRSA isolates. Chemical analysis identified β‐pinene (38.73%) and α‐pinene (11.79%) as the major constituents of EOAM. The oil exhibited synergistic effects when combined with amoxicillin and oxacillin, inhibiting biofilm formation and reducing *S. aureus*‐induced hemolysis. Additionally, EOAM enhanced the efficacy of hydrogen peroxide (H₂O₂) against *S. aureus*. In vivo, EOAM significantly increased the survival of *Galleria mellonella* larvae infected with MRSA, reducing bacterial load and myelinization. These findings highlight the therapeutic potential of EOAM in treating MRSA infections by modulating bacterial resistance and virulence, suggesting their potential for application in clinical settings.

## Graphical abstract

This study explored the antibacterial, antivirulence, and in vivo effects of *Algrizea minor* essential oil (EOAM), against MRSA isolates. Chemical analysis by GC‐MS has been performed. The oil exhibited synergistic effects when combined with amoxicillin and oxacillin, inhibiting biofilm formation and reducing S. aureus‐induced hemolysis. EOAM enhanced the efficacy of hydrogen peroxide (H_2_O_2_) against *S. aureus. In vivo*, EOAM significantly increased the survival of *Galleria mellonella* larvae infected with MRSA, reducing bacterial load and myelinization.

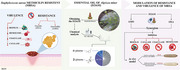

## 1 Introduction

Methicillin‐resistant Staphylococcus aureus (MRSA) strains have become a public health emergency, as these isolates have several metabolic adaptation mechanisms, such as toxin production and enzyme secretion, which allow them to evade the host's defenses and persist within the body more effectively. MRSA is also capable of forming biofilms on artificial and biological surfaces, which makes treatment difficult when this pathogen is associated with severe skin infections, endocarditis, and pneumonia [1, 2, 3].

With the global spread of MRSA isolates, coupled with a decline in the effective therapeutic options available, there has been a significant increase in morbidity and mortality worldwide [4, 5]. The effectiveness of traditional antibiotics is limited to their bactericidal or bacteriostatic effects, to which bacteria can easily acquire resistance, making them less effective over time. The reduction in therapeutic options available for multidrug‐resistant strains such as MRSA, combined with the presence of virulence mechanisms, increases the difficulty of treatment. It is therefore essential to develop alternative therapeutic strategies that identify multifactorial active compounds to effectively combat MRSA infections [6, 7].

Considering the significant time and financial investment required to develop new antimicrobials, a promising alternative is the use of molecules that are synergistic with established clinical antibiotics. In the search for new therapeutic alternatives, essential oils (EOs), especially those rich in terpenes, have demonstrated the potential to disrupt bacterial cell membranes, inhibit efflux pumps, and modulate gene expression. Consequently, EOs are emerging as a promising alternative due to their potential to act as agents that modulate bacterial resistance and virulence [8, 9].

It is notable that plants from the Caatinga phytogeographic domain produce EOs with a wide range of biological activities and are considered valuable sources of bioactive molecules. The Caatinga is an exclusively Brazilian domain with unique characteristics, which allows its EOs to present exclusive combinations of secondary metabolites. As they are complex mixtures of metabolites, they can act synergistically to generate different biological effects. Algrizea minor Sobral, Faria & Proença (Myrtaceae) is a shrub endemic to the Caatinga [10]. Despite the limited number of studies exploring the biological activities of this plant, its chemical profile, characterized by a complex mixture of terpenes, suggests potential for investigating its antibacterial and resistance‐modulating properties against MRSA isolates.

This study aims to investigate the antibacterial activity of *Algrizea minor* EO (EOAM) and its potential to modulate resistance and virulence in MRSA isolates, particularly in combination with conventional antibiotics. By exploring these multifactorial applications, the research attempts to identify new therapeutic strategies that utilize the chemical profile of EOAM to combat antibiotic‐resistant pathogens.

## 2 Methodology

### 2.1 Collection and Extraction of EO

The leaves of *A. minor* were collected in the Vale do Catimbau National Park, in the municipality of Buíque‐PE (8° 30′ 57’’ S 37° 20′ 59’ W), in September 2022. The material was processed and compared with a specimen deposited in the Herbarium Professor Vasconcelos Sobrinho (PEUFR), with the registration number PEUFR35193, for species confirmation. After collection, 500 g of fresh plant material was subjected to hydrodistillation in a Clevenger apparatus for 4 h at 96°C to extract the Essential Oil (EO). Once obtained, the EO was dried with sodium sulfate (Na_2_SO_4_) and stored in amber vials at 4°C. The yield (w/w) was then determined using the following formula: *To* = (*Vo*/*Bm*) × 100, where the content (*To*) of EO on a wet basis is determined by the relationship between the volume of oil (*Vo*) and the plant biomass (*Bm*) [11].

### 2.2 Analysis of the Chemical Profile of EO

Analysis of the chemical profile was performed using gas chromatography‐mass spectrometry (GC‐MS). The equipment used for the GC‐MS analyses was a quadrupole Agilent, model 5977B, equipped with a non‐polar fused silica HP‐5 ms capillary column (Agilent J&W) (30 m × 0.25 mm i.d.; film thickness 0.25 mm). Helium was used as the carrier gas at a total flow rate of 0.225 L/min. Temperatures were set and maintained at 250°C for the injector and 280°C for the detector. The oven was programmed at an initial temperature of 40°C for 1 min with a progressive increase of 6°C/min until 300°C was reached and maintained for 6 min. Approximately 1 µL of sample was injected individually into the column using the injector at a split ratio of 1:200. The ionization potential was 70 eV, while the scan range was 40 to 550 m/z at a rate of 0.5 scans per second. The calculated indices for each compound were compared with the corresponding values reported in the literature [12]. The relative amounts of the identified compounds were determined by gas chromatography coupled to a flame ionization detector (GC‐FID). The instrument used for the analyses was a TRACE GC Ultra (Thermo Scientific) equipped with a non‐polar DB‐5 column (60 m × 0.25 mm i.d.; 0.25 mm film thickness), using the same analytical conditions as for GC‐MS.

### 2.3 Microbial Material

Isolates of *S. aureus* ATCC 6538 (Methicillin‐resistant *Staphylococcus aureus* [MRSA]) and ATCC 29213 were obtained from the collection of the Molecular Biology Laboratory (BioMol) of the Biochemistry Department of the Federal University of Pernambuco (UFPE), and *S. aureus* UFPEDA 20 (MRSA) was obtained from the Collection of Mycroorganisms of the Department of Antibiotics of UFPE (UFPEDA). The isolates were reactivated with overnight growth in brain heart infusion (BHI) broth. Bacterial suspensions were prepared with a 0.9% sodium chloride (NaCl) solution, and the inoculum was adjusted to 0.5 McFarland using a spectrophotometer at 600 nm.

### 2.4 Determination of the Minimum Inhibitory Concentration

The determination of the minimum inhibitory concentration (MIC) of EOAM was performed according to the recommendations of the Clinical and Laboratory Standards Institute (CLSI) [13]. Assays were performed by serial microdilution in 96‐well microplates, and the plates were incubated at 37°C for 24 h The MIC was defined as the lowest concentration that resulted in visible growth inhibition after the addition of 20 µL of 0.04% sodium resazurin solution. Mueller‐Hinton (MH) broth with and without inocula was maintained as positive and negative controls, respectively.

### 2.5 Assessment of the Modulatory Effect

The evaluation was carried out using the checkerboard method described in the literature [14]. In a microdilution plate, 50 µL of MH medium and 50 µL of the MICs of EOAM and the antibiotics oxacillin (OXA), azithromycin (AZT), amoxicillin (AMO), and cephalexin (CEF) were added in a standard checkerboard layout. After incubation at 37°C for 24 h, visible growth was observed after the addition of 0.04% resazurin, and the Fractional Inhibitory Concentration Index (FICI) was calculated by dividing the MIC of the combined treatment by the MIC of the individual treatment. The sum of the FICs (ΣFIC) was calculated, and interactions were considered synergistic when the FICI index was ≤0.5, additive when 0.5< FICI ≤ 1, indifferent when 1< FICI ≤ 2, and antagonistic interactions when FICI ≥ 2. The effect of the MIC of the EOAM and the synergistic concentrations obtained in the checkerboard tests were evaluated in all tests. MH broth with and without inocula was maintained as positive and negative controls, respectively.

### 2.6 Kill Curve

Bacterial elimination kinetics were evaluated using the protocol described by Kumari et al. [15], with modifications. Bacterial suspensions were prepared in microtiter plates at 0.5 McFarland and incubated in an oven at 37°C for 8 h to reach the logarithmic growth phase. The treatments were then added, and the plates were incubated for 12 h in a Multiskan FC plate reader (Thermo Scientific) at 37°C and read every h at 600 nm to evaluate the reduction in bacterial load and the bactericidal effects of the treatments. MH broth with and without inocula was kept as positive and negative controls, respectively.

### 2.7 Assays for Antivirulence Activity

#### 2.7.1 Antibiofilm Activity

Biofilm formation by *S. aureus* in response to the treatments was evaluated using the crystal violet (CV) uptake quantification method. After 24 h of incubation at 37°C, the microtiter plates were washed three times with 0.9% NaCl saline to remove non‐adherent cells. The biofilm was then fixed at 55°C for 1 h, followed by the addition of 0.04% CV for 15 min. The plates were washed again with saline, and 99% ethanol was added for 30 min. After this period, readings were taken at a wavelength of 570 nm [16]. MH broth with and without inocula was maintained as positive and negative controls, respectively.

#### 2.7.2 Hydrogen Peroxide Sensitivity

The method described by Bezerra‐Filho et al. [17] was used with modifications to evaluate susceptibility to H_2_O_2_. First, a microbial suspension was prepared from overnight cultures of *S. aureus* and seeded as a lawn on Petri plates containing MH agar (HIMEDIA). Filter paper discs (6 mm) were impregnated with H_2_O_2_ (1.5%, v/v), distilled water (H_2_O_2_), and treatments with the MIC of EOAM and its synergistic concentrations with antibiotics. Plates were incubated at 37°C for 24 h. Susceptibility to H_2_O_2_ was assessed by measuring the halo formed by the association of treatments.

#### 2.7.3 Coagulase Assay

The effect of treatments on the inhibition of *S. aureus*‐induced coagulation was evaluated by the method described by Xiang et al. [18]. In Eppendorf tubes, 150 µL of bacterial suspensions with treatments were prepared in BHI broth and incubated at 37°C for 24 h. Then, 100 µL of the tubes were resuspended in 250 µL rabbit blood plasma (Coagu‐plasma, Laborclin) and incubated at 37°C for 4 h. MRSA without treatments was used as a positive control (CTL +) and EOAM without bacteria as a negative control (CTL ‐). The inhibition of clot formation was visually graded as: complete (no consistency or resistance to tube inversion), partial (low consistency and unable to resist tube inversion), and absent (firm and resistant to tube inversion).

#### 2.7.4 Hemolysis Assay

For the hemolysis assay, bacteria were inoculated in BHI with the treatments at a 1:100 (v/v) ratio. After 16 h, 500 µL of the cultures were added to 1 mL of mouse erythrocyte solution (3%) (ethics committee: 0053/20) and incubated at 37°C for 2 h. The supernatant was collected after centrifugation at 3000 × *g* for 10 min, and the optical density (OD) was measured at 540 nm. The antihemolytic effect was evaluated according to the formula: Hemolysis rate (%) = [(absorbance of sample – absorbance of negative control)/(absorbance of positive control – absorbance of negative control)] × 100%. Saponin and saline (0.85% NaCl) were used as positive and negative controls, respectively [17].

### 2.8 Evaluation of in Vivo Antibacterial Activity

The *Galleria mellonella* model was selected due to its practical advantages over vertebrate models, including low cost, ease of handling, and absence of ethical restrictions. This invertebrate has an innate immune system functionally similar to that of mammals, with hemocytes and immune pathways capable of responding to bacterial infections.

#### 2.8.1 Toxicity Assay

For the toxicity assay, 10 *G. mellonella* larvae (average weight 200 mg) between the fifth and sixth instar (2–3 cm) were used per group. Initially, 10 µL of the treatments were applied to the last left proleg of each larva. The larvae were then maintained at 37°C for 5 days, and survival was assessed every 24 h.

#### 2.8.2 Survival Rate

Larvae were infected in the last proleg with 10 µL of bacterial inoculum adjusted to 1 McFarland. After 30 min, 10 µL of the treatments at the MIC of EOAM and the synergistic concentrations were administered to the last proleg. The groups were incubated at 37°C for 5 days, and larval survival was evaluated every 24 h [19].

#### 2.8.3 Assessment of Bacterial Load in Hemolymph

After 24 and 48 h, 10 µL of hemolymph was collected from treated and untreated larvae and diluted 1:10 in phosphate buffered saline (PBS) (white). The PBS solution was also used as a control in the untreated groups. Approximately 10 µL of the dilutions were plated on mannitol salt agar for selection and differentiation of *S. aureus*. Plates were incubated at 37°C to quantify the microbial load in CFU/mL [17].

#### 2.8.4 Determination of Hemocyte Viability

Twenty microliters of hemolymph was collected from treated and untreated larvae after infection with *S. aureus*, and 20 µL of 0.02% trypan blue in PBS (white) was added. Using a Neubauer chamber, cell viability was observed based on the ability of hemocytes to exclude trypan blue from the intracellular space [17].

#### 2.8.5 Quantification of Melanization

To quantify melanization, a solution of 20 µL of larval hemolymph with 80 µL of PBS (white) was added to microdilution plates. After 5 min, the OD was observed in a spectrophotometer at 405 nm [19].

### 2.9 Statistical Analysis

All the tests described were carried out in triplicate. Data were analyzed using GraphPad Prism software (version 8.0.2). Statistical analyses were performed using one‐way and two‐way analysis of variance (ANOVA), with Tukey's and Sidak's tests, at a significance level of 95% (*p* ≤ 0.05). Larval survival curves were determined using the log‐rank test (Mantel‐Cox).

## 3 Results and Discussion

EOAM yielded 0.54% (w/w), identifying 31 compounds, of which 64.52% were sesquiterpenes and 35.48% were monoterpenes. The secondary metabolites β‐pinene (38.73%) and α‐pinene (11.79%) were the most abundant compounds (Table 1), corroborating the results found by Fernandes et al. [20] and Veras et al. [21], who also observed β‐pinene and α‐pinene as the majority compounds. The diversity of terpenes in the composition of EOAM confirms the common chemical signature among plants of the Myrtaceae family, which possess a variety of terpene synthase enzymes.

**TABLE 1:** Chemical composition of essential oil of *Algrizaea minor*.

Compound	CKI	LKI	%
α‐Thujene	924	924	0.54
α‐Pinene	930	932	11.79
β‐Pinene	972	974	38.73
Myrcene	985	988	0.68
δ‐3‐Carene	1005	1008	0.21
ρ‐Cymene	1019	1020	1.84
Sylvestrene	1024	1025	3.65
Linalool	1095	1095	0.28
endo‐Fenchol	1108	1114	0.61
trans‐Pinocarveol	1134	1135	0.29
Pinocarvone	1158	1160	0.13
Borneol	1161	1165	0.07
Terpinen‐4‐ol	1173	1177	2.01
α‐Terpineol	1186	1186	1.83
Myrtenol	1192	1194	0.63
α‐Ylangene	1369	1373	0.12
α‐Copaene	1374	1374	0.29
β‐Elemene	1390	1389	0.77
α‐Gurjunene	1408	1409	0.13
*(E)*‐Caryophyllene	1418	1417	4.47
Aromadendrene	1439	1439	0.54
α‐Humulene	1452	1452	1.57
Aromadendrene	1459	1458	0.57
γ‐Muurolene	1474	1478	0.48
Germacrene D	1479	1484	2.38
β‐Selinene	1484	1489	0.42
Bicyclogermacrene	1494	1500	2.90
α‐Muurolene	1497	1500	0.08
Germacrene A	1503	1508	0.14
δ‐Cadinene	1520	1522	0.83
Hedycaryol	1546	1546	1.29
		Total	96.55

*Note*: CKI, Calculated Kovats Index; LKI, Literature Kovats Index; Relative percentage of compounds (%).

EOAM has few studies on its biological activities, which are attributed to analgesic, antioxidant, gastroprotective, and antimicrobial activities [22, 23]. Terpenes and their derivatives have bacteriostatic and bactericidal effects on various pathogens, including those with antimicrobial resistance (AMR), mainly by disrupting the cell membrane or inhibiting molecular synthesis [22].

EOAM showed anti‐*S. aureus* activity at concentrations of 32 µg/mL and 8 µg/mL for ATCC 25923 and ATCC 6538, respectively. Similar results were found by Veras et al. [21], where EOAM had a MIC of 32.5 µg/mL for *S. aureus* ATCC 6538. For *S. aureus* UFPEDA 20, the MIC of the EO was 2048 µg/mL (Table 2). UFPEDA 20 was resistant to β‐lactam antibiotics and macrolides, while ATCC 6538 and 25923 were susceptible to all antibacterial agents according to the parameters established by CLSI [13]. In order to evaluate the possible synergistic effect and the reduction in resistance of UFPEDA 20 (MRSA) to the antibiotics tested, a total of 76 combinations of different concentrations of EOAM associated with antibiotics were analyzed using the checkerboard method (Table 2).

**TABLE 2:** Susceptibility profile and evaluation of the minimum inhibitory concentration of *Algrizea minor* essential oil on *Staphylococcus aureus* strains (µg/mL).

Stain	Antibiotics				OEAM
	OXA	AZT	AMO	CEF	
UFPEDA 20	8	16	4	16	2048
ATCC 6538	0.5	2	0.5	1	32
ATCC 25923	0.5	1	0.25	4	8

Abbreviations: AMO, amoxicillin; AZT, azithromycin; CEF, cephalexin; EOAM, *Algrizea minor* essential oil; OXA, oxacillin.

This research corresponds to the first report on the modulatory effect of EOAM associated with antibiotics to reverse resistance. When combined with 64 and 128 µg/mL EOAM, the MICs of AMO and OXA were reduced from 4 and 8 µg/mL, respectively, to 0.12 µg/mL. The combinations resulted in a significant synergistic effect with FICI of 0.06 and 0.07, respectively. In the *Eucalyptus citriodora* (Myrtaceae) EO analyzed by Pinheiro et al. [24], the authors observed a synergistic effect with antibiotics against MRSA, with the most significant MIC reductions for gentamicin (from 250 to 19.67 µg/mL) and ciprofloxacin (from 78.75 to 0.50 µg/mL).

The synergistic association of EOs with antibiotics is beneficial because EOs contain different secondary metabolites that can act in different ways to potentiate the antibiotic effect. The EO of *Syzygium cumini* (Myrtaceae) analyzed by Fernandes et al. [24] had α‐pinene (53.21%) as the main component and demonstrated possible synergy when associated with gentamicin in *S. aureus*, reducing the MIC of gentamicin from 30 to 10 µg/mL.

In the present study, the synergistic effects between EOAM occurred mainly with β‐lactam antibiotics. Saraiva et al. [14] also reported that the best synergistic indices were observed with the EO of *Laurus nobilis* (Lamiaceae) in association with β‐lactam antibiotics. Historically, β‐lactams have a slow bactericidal action, so modifications to the original molecule lead to the formation of new compounds, changes in spectrum and activity [25]. Bacteria classified as MRSA have resistance to these antibiotics, which is regulated by the expression of penicillin‐binding protein 2. Therefore, only a significant proportion of MRSA strains are susceptible to penicillins, but only when associated with β‐lactamase inhibitors [26].

**FIGURE 1:**
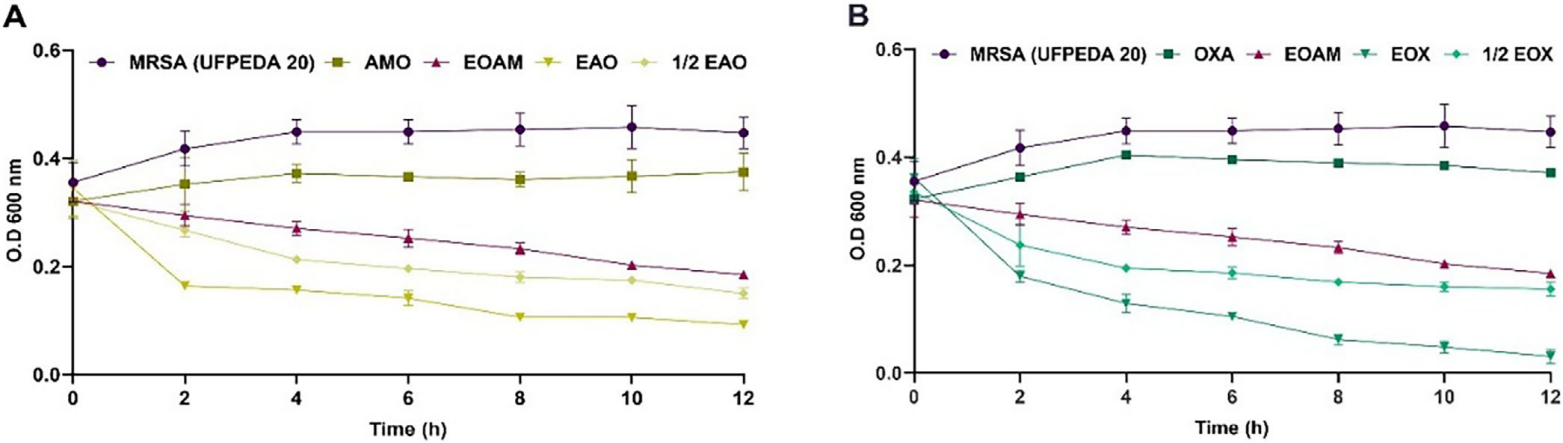
Methicillin‐resistant *Staphylococcus aureus* (MRSA) death kinetics against *Algrizea minor* essential oil (EOAM), amoxicillin (AMO), and oxacillin (OXA) alone and in their respective synergistic concentrations EAO (A) and EOX (B), and sub‐synergistic concentrations (½ EAO and ½ EOX). Legend: Concentrations of the compounds used were recent to the minimum inhibitory concentrations (MICs) of EOAM (2048 µg/mL), AMO (4 µg/mL), and OXA (8 µg/mL), alone, as well as the combinations EAO (64 µg/mL of EOAM and 0.12 µg/mL AMO), ½ EAO (32 µg/mL EOAM and 0.06 µg/mL AMO), EOX (128 µg/mL EOAM and 0.12 µg/mL OXA), and ½ EOX (64 µg/mL EOAM and 0.06 µg/mL OXA).

The antibiotics OXA and AMO did not significantly reduce the bacterial load in the elimination curve compared to the positive control (*p* > 0.9999), while the MIC of EOAM caused a reduction in bacterial load of approximately 49.3% (Figure 1). However, in the first 2 h, the synergistic concentrations of EOAM with AMO (EAO) (Figure 1A) and OXA (EOX) (Figure 1B) resulted in a reduction of 53.8% and 49.57%, reaching a reduction of 74.08% and 91.55% after 12 h, respectively. These results indicate that synergy is time‐dependent, with EO improving the performance of the antibiotic.

In the elimination kinetics conducted by Yang et al. [27], the authors also observed that the isolated use of *Lavandula* sp. (Lamiaceae) EO and meropenem did not affect the viability of carbapenemase‐producing *Klebsiella pneumoniae* (KPC). However, when combined, the two compounds achieved a complete *K. pneumoniae* elimination profile (FICI = 0.31), indicating that the combination improved performance in reducing the bacterial load. The *Pelargonium endlicherianum* (Geraniaceae) EO analyzed by Dumlupinar et al. [28] did not effectively reduce the number of *K. pneumoniae* bacterial cells compared to the EO combined with gentamicin and cefepime, resulting in a significantly faster reduction in the number of live cells after the sixth h.

**FIGURE 2:**
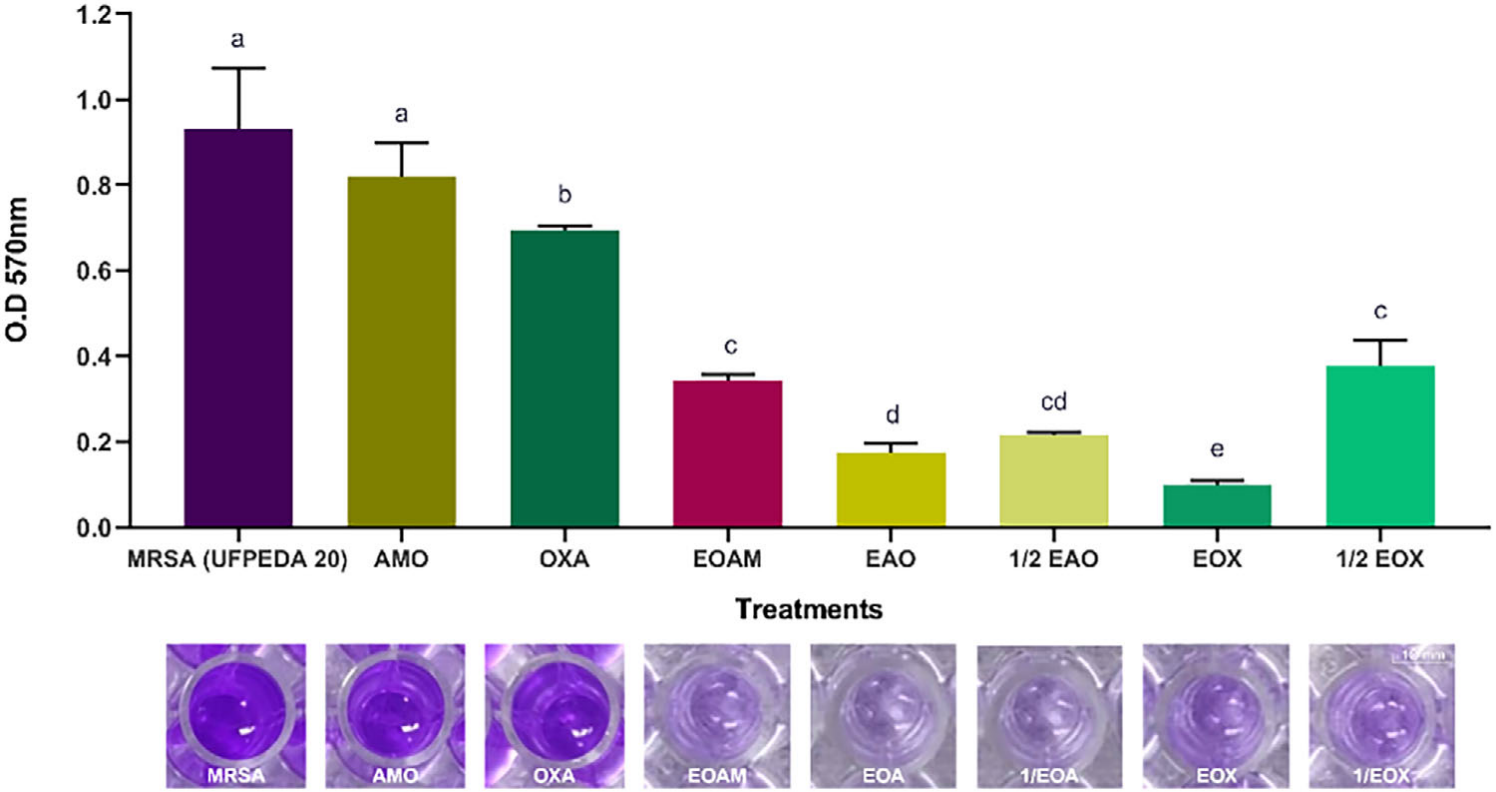
Antibiofilm activity of *Algrizea minor* essential oil (EOAM), amoxicillin (AMO), and oxacillin (OXA) alone and in their respective synergistic concentrations EAO and EOX, and subsynergistic concentrations (½ EAO and ½ EOX) on methicillin‐resistant *Staphylococcus aureus* (MRSA) (UFPEDA 20). Legend: Bacterial growth without treatment (Control) was used as a reference. Different lowercase letters represent significant differences between treatments at the same time, according to the Tukey test (*p <* 0.05). Concentrations of the compounds used were recent to the minimum inhibitory concentrations (MICs) of EOAM (2048 µg/mL), AMO (4 µg/mL), and OXA (8 µg/mL), alone, as well as the combinations EAO (64 µg/mL of EOAM and 0.12 µg/mL AMO), ½ EAO (32 µg/mL EOAM and 0.06 µg/mL AMO), EOX (128 µg/mL EOAM and 0.12 µg/mL OXA), and ½ EOX (64 µg/mL EOAM and 0.06 µg/mL OXA).

*S. aureus* is a pathogen with different virulence mechanisms that are regulated by complex networks sensitive to environmental signals and interactions with the host, which makes treatment difficult. AMO (p = 0.2525) failed to inhibit MRSA biofilm formation, while OXA inhibited only 24.44%. However, EOAM showed an inhibition of 63.12%, which was not significantly different from the treatments with concentrations of ½ EOA (p = 0.1510) and ½ EOX (p = 0.9506) (Figure 2). Although EO has an antibiofilm effect, inhibition is enhanced when EOAM is associated with antibiotics at lower concentrations.

For EOA (Figure 2A) and EOX (Figure 2B), the antibiofilm effects were more significant, with inhibition rates of 81.14% (*p* < 0.0001) and 89.32% (*p* < 0.0001), respectively. Similarly, Rosato et al. [29] described a positive effect of combining *Cinnamomum zeylanicum* (Lauraceae) EO (600 µg/mL) with OXA (7.7 µg/mL) on *S. aureus* (ATCC 29213), in which the synergistic concentration reduced biofilm formation by 67.2%.

**FIGURE 3:**
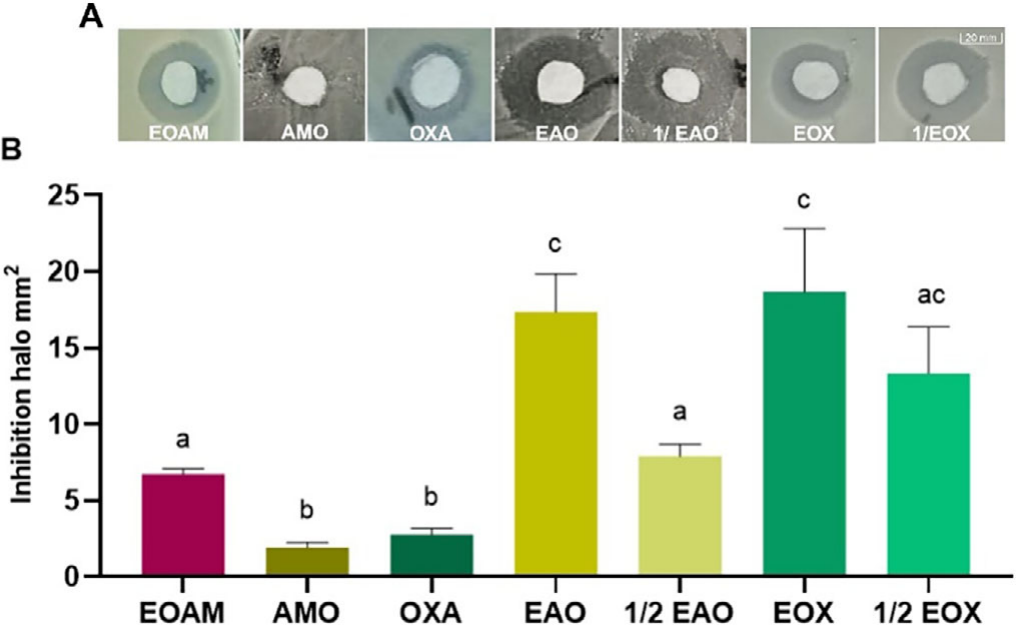
Evaluation of the susceptibility of methicillin‐resistant *Staphylococcus aureus* (MRSA) (UFPEDA 20) (A) to hydrogen peroxide (1.5%) against the effect of *Algrizea minor* essential oil (EOAM), amoxicillin (AMO), and oxacillin (OXA) alone and in their respective synergistic concentrations EAO and EOX (B), and subsynergistic concentrations (½ EAO and ½ EOX). Legend: The phenotypic analysis of inhibition of peroxidase activity was assessed by measuring the inhibition halo of the treatments alone and in synergistic concentrations. Different lowercase letters represent significant differences between treatments at the same time, according to the Tukey test (*p* < 0.05). Concentrations of the compounds used were recent to the minimum inhibitory concentrations (MICs) of EOAM (2048 µg/mL), AMO (4 µg/mL), and OXA (8 µg/mL), alone, as well as the combinations EAO (64 µg/mL of EOAM and 0.12 µg/mL AMO), ½ EAO (32 µg/mL EOAM and 0.06 µg/mL AMO), EOX (128 µg/mL EOAM and 0.12 µg/mL OXA), and ½ EOX (64 µg/mL EOAM and 0.06 µg/mL OXA).

Another important virulence factor is the action of the enzyme catalase, which protects the *S. aureus* biofilm from the lethality of reactive oxygen species (ROS), thus helping to maintain the biofilm [30]. As well as facilitating cell detoxification, the enzyme repairs or prevents oxidative damage caused by H_2_O_2_ [31]. In the present study, H_2_O_2_ (1.5% v/v) was unable to induce oxidative stress in MRSA, with no halo of inhibition observed on the control disk. In the presence of EOAM alone or in association with antibiotics, H_2_O_2_ showed a significant toxic effect (*p* < 0.0001), in which treatments with EOA and EOX increased the inhibition halo by 61.3% and 49.62%, respectively. This indicates that treatments can act both to prevent biofilm formation and to protect against it (Figure 3). In a study carried out by Bezerra‐Filho et al. [17], the EO of *Eugenia brejoensis* Mazine at 64 µg/mL potentiated the toxicity of H_2_O_2_ by 24.12%.

The coagulase enzyme also plays a fundamental role in the pathogenesis of infections caused by *S. aureus*, promoting blood clotting and helping to establish bacterial colonies in the host's tissues [32]. However, small molecules can bind to proteins, induce conformational changes, and alter their properties [18]. At the MIC concentration, EOAM completely inhibited clot formation induced by *S. aureus* (Table 3). Total inhibition of coagulase was also observed due to the effect of EOA and EOX, as well as at the concentration of ½ EOX. As EOs are composed of different molecules, secondary metabolites with different chemical structures can interact with the proteins that make up the coagulase enzyme, allowing conformational changes and inactivating their effect.

**TABLE 3:** Effect of treatments on the inhibition of the coagulase enzyme produced by methicillin‐resistant *Staphylococcus aureus* (MRSA) (UFPEDA 20) of *Algrizea minor* essential oil (EOMA), amoxicillin (AMO), and oxacillin (OXA), and their respective synergistic associations EAO (A) and EOX (B), and subsynergistic (½ EAO and ½ EOX).

Strain		Controls				Synergistic concentrations			
	CTL ‐	CTL +	EOAM	AMO	OXA	EAO	½ EAO	EOX	½ EOX
UFPEDA 20	++	—	++	—	—	++	+	++	++

*Note*: Total (++); Partial (+); Absent (‐); EOAM without bacteria (CTL ‐); MRSA (CTL +); Concentrations of the compounds used were recent to the MICs of EOAM (2048 µg/mL), AMO (4 µg/mL), and OXA (8 µg/mL), alone, as well as the combinations EAO (64 µg/mL of EOAM and 0.12 µg/mL AMO), ½ EAO (32 µg/mL EOAM and 0.06 µg/mL AMO), EOX (128 µg/mL EOAM and 0.12 µg/mL OXA), and ½ EOX (64 µg/mL EOAM and 0.06 µg/mL OXA).

EOs have significant anticoagulant properties, which could be beneficial for the development of therapies based on natural anticoagulants. Some authors have described that terpene‐rich EOs have anticoagulant activity. Drioiche et al. [33] demonstrated that the EO of *Pistacia lentiscus* (Anacardiaceae) at concentrations of 2.875 mg/mL and 5.750 mg/mL exhibited anticoagulant activity by inhibiting endogenous and exogenous coagulation pathways in a dose‐dependent manner. Terpenes can influence anticoagulant activity by interacting with factors in the blood coagulation system, acting to inhibit platelet aggregation, reducing thrombus formation, and the activity of enzymes involved in coagulation [34].

**FIGURE 4:**
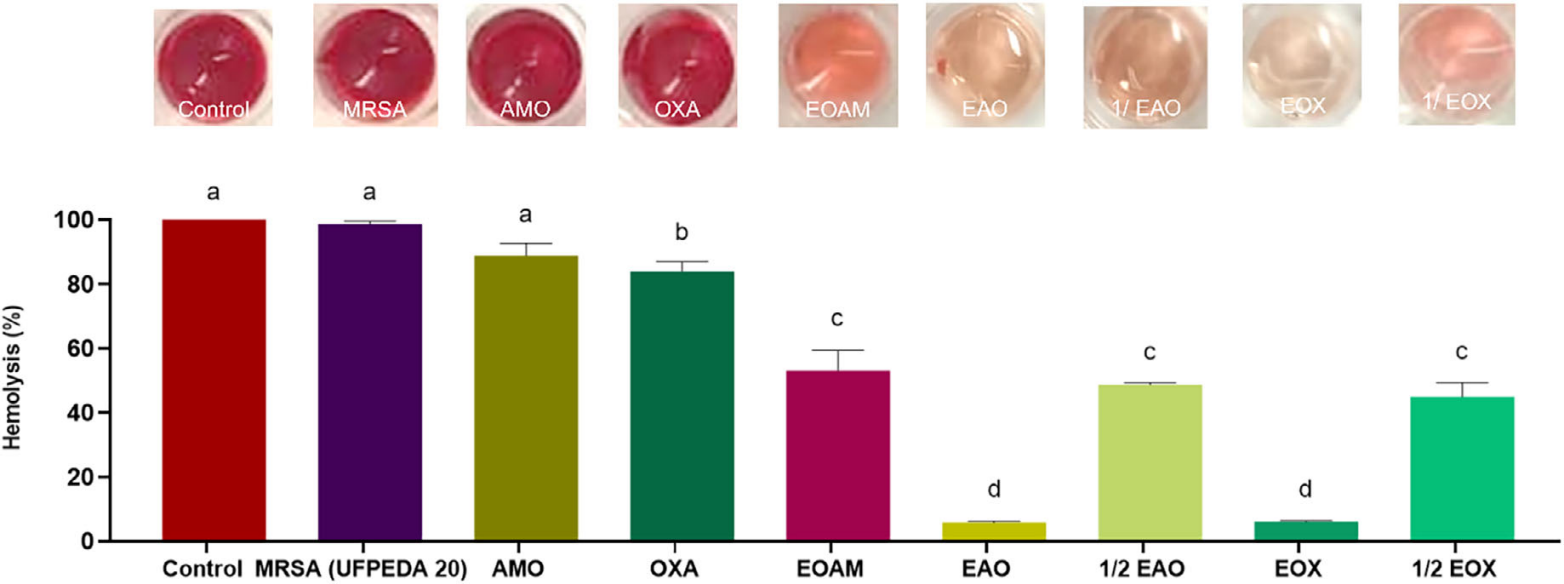
Anti‐hemolytic effect of *Algrizea minor* essential oil (EOMA), amoxicillin (AMO), and oxacillin (OXA) and their respective synergistic associations EAO and EOX, and sub‐synergistic (½ EAO and ½ EOX). Legend: Saponin was used as a reference for 100% hemolysis (Control), and the effectiveness of the treatments was compared with the hemolysis caused by the methicillin‐resistant *Staphylococcus aureus* (MRSA) strain (*S. aureus* UFPEDA 20). Different lowercase letters represent significant differences between treatments at the same time, according to the Tukey test (*p* < 0.05). Concentrations of the compounds used were recent to the minimum inhibitory concentrations (MICs) of EOAM (2048 µg/mL), AMO (4 µg/mL), and OXA (8 µg/mL), alone, as well as the combinations EAO (64 µg/mL of EOAM and 0.12 µg/mL AMO), ½ EAO (32 µg/mL EOAM and 0.06 µg/mL AMO), EOX (128 µg/mL EOAM and 0.12 µg/mL OXA), and ½ EOX (64 µg/mL EOAM and 0.06 µg/mL OXA).

Some natural compounds also have a significant inhibitory effect on the hemolytic activity of *S. aureus*. Bezerra‐Filho et al. [17] observed that subinhibitory concentrations of *E. brejoensis* EO inhibited *S. aureus*‐mediated hemolysis by approximately 90%. Compared to the hemolytic activity of *S. aureus*, EOAM reduced hemolysis by 46.99% (*p* < 0.0001). In synergistic concentrations with the antibiotics AMO and OXA, the anti‐hemolytic effect exhibited rates of up to 94.4% (*p* < 0.0001) (Figure 4).

Similarly, Rubini et al. [35] demonstrated the efficacy of *Pogostemon heyneanus* (Lamiaceae) and *Cinnamomum tamala* (Lauraceae) EOs in reducing the hemolytic activity of *S. aureus* MRSA strains by 90% and 65%, respectively. The authors describe that hemolysin production is not only a vital virulence factor, but also facilitates biofilm formation, suggesting that EOs act on different *S. aureus* virulence mechanisms.

**FIGURE 5:**
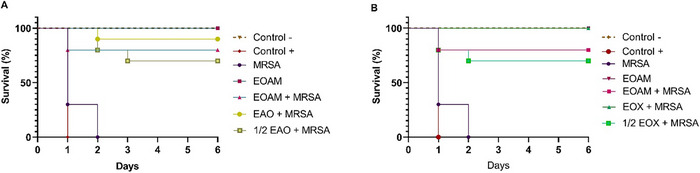
Survival curve of *Galleria mellonella* in an infection model induced by methicillin‐resistant *Staphylococcus aureus* (MRSA) (UFPEDA 20) and with the treatment of *Algrizea minor* essential oil (EOMA), and its synergistic associations EAO (A) and EOX (B), sub‐synergistic (½  EAO and ½  EOX). Legend: Phosphate‐buffered saline solution (PBS) was used as a negative control (CTL ‐), while dimethyl sulfoxide served as a positive control (CTL +). The essential oil (EOAM) was evaluated without the bacterial inoculum to assess toxicity. Subsequently, the essential oil and its synergistic and subsynergistic cencontractions with the antibiotics were applied to assess larval survival after infection with the MRSA strain. Concentrations of the compounds used were recent to the minimum inhibitory concentrations (MICs) of EOAM (2048 µg/mL), AMO (4 µg/mL), and OXA (8 µg/mL), alone, as well as the combinations EAO (64 µg/mL of EOAM and 0.12 µg/mL AMO), ½ EAO (32 µg/mL EOAM and 0.06 µg/mL AMO), EOX (128 µg/mL EOAM and 0.12 µg/mL OXA), and ½ EOX (64 µg/mL EOAM and 0.06 µg/mL OXA).

The MIC treatments of EOAM, EOA, and EOX showed no toxicity in the in vivo model of *G. mellonella*, with 100% survival. After infection with *S. aureus*, EOA and EOX showed a significant effect on larval survival compared to the untreated control group (*p* < 0.0001) (Figure 5). Synergistic concentrations increased the survival rate up to 100%, while larvae with sub‐synergistic concentrations of ½ EAO and ½ EOX showed a survival rate of 70%. Without treatment, mortality reached 100% after 3 days of the experiment, with a significant increase in the bacterial load of *S. aureus* detected in the hemolymph between 24 and 48 h (*p* < 0.0001).

The treated larvae were able to reduce the bacterial load in the hemolymph by more than 98% (Table 4). The treatments with EOA (*p* = 0.4630), EOX (0.7168), and ½ EOX (>0.9999) showed no significant increase in bacterial load within the experimental interval, indicating that the treatments confer stability to the antibacterial effect.

**TABLE 4:** Reduction in the bacterial load of methicillin‐resistant *Staphylococcus aureus* (MRSA) (UFPEDA 20) in the hemolymph of *Galleria mellonella* after treatment with synergistic concentrations of *Algrizea minor* essential oil with amoxicillin (EAO) and oxacillin (EOX).

Treatments	UFC /mL	
	24 h	48 h
Control (MRSA)	4.374 ±52^a^	4.901 ± 57^a^
EAO	35 ± 7^a^	17 ± 5^a^
½ EAO	1146 ± 26^a^	1248 ±29^a^
EOX	65 ± 7^a^	51 ± 11^a^
½ EOX	1649 ± 59^a^	1692 ± 33^a^

*Note*: Different lowercase letters represent significant differences between the analysis times of the treatments according to the Sidak test (*p* < 0.05). Concentrations of the compounds used were recent to the MICs of EOAM (2048 µg/mL), AMO (4 µg/mL), and OXA (8 µg/mL), alone, as well as the combinations EAO (64 µg/mL of EOAM and 0.12 µg/mL AMO), ½ EAO (32 µg/mL EOAM and 0.06 µg/mL AMO), EOX (128 µg/mL EOAM and 0.12 µg/mL OXA), and ½ EOX (64 µg/mL EOAM and 0.06 µg/mL OXA).

Alnezary et al. [1] found positive effects in the combination of EOs and antibiotics in the treatment of MRSA‐induced infections in *G. mellonella*. The authors described that the combination of vancomycin (20 mg/kg) with *Nigella sativa* (Ranunculaceae) EO (70 mg/kg) contributed to the survival of 73% of the individuals, with lower survival rates than those presented in the present study.

**FIGURE 6:**
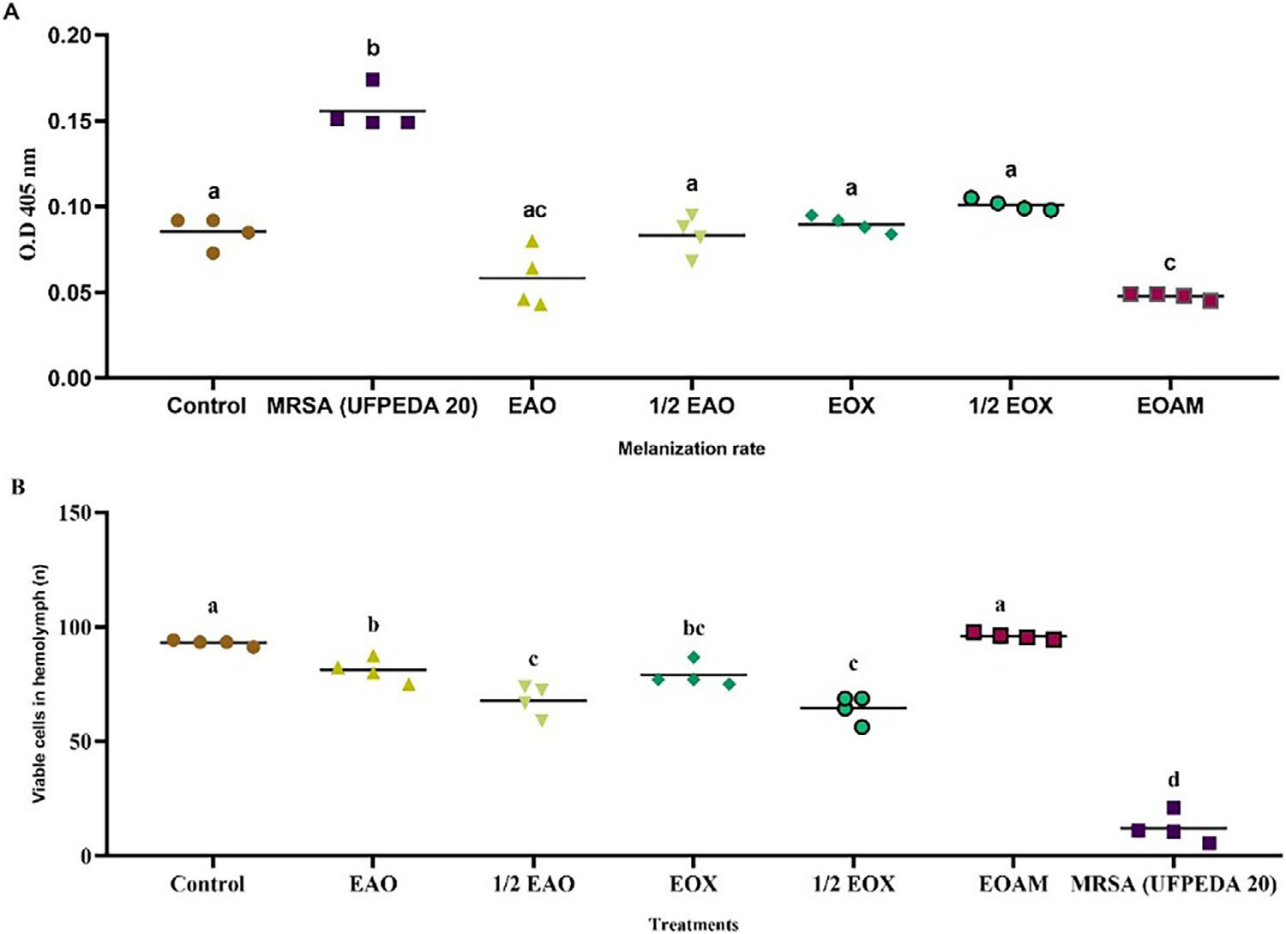
Evaluation of the effect of synergistic concentrations of *Algrizea minor* essential oil (EOAM) with Amoxicillin (EAO) and Oxacillin (EOX), and subsynergistic (½ EAO and ½ EOX), on melanization (A) and hemocyte viability (B) in the hemolymph of *Galleria mellonella* after infection with methicillin‐resistant *Staphylococcus aureus* (MRSA) (UFPEDA 20). Legend: PBS (Control); The rate of melanization and the viability of hemocytes were also evaluated in uninfected groups, applied only with the essential oil of *Algrizea minor* (EOAM), to evaluate the effect of the oil on the immune system of the wash; Also, larvae infected with *S.aureus*, and which did not receive treatment, were evaluated during the period of the experiment. After infection with *S. aureus*, synergistic concentrations of the essential oil were applied with the antibiotics Amoxicillin (EAO) and Oxacillin (EOX), in order to assess the protection of the treatments against infection by a pathogen. Different lowercase letters represent significant differences between treatments at the same time, according to the Tukey test (*p* < 0.05). Concentrations of the compounds used were recent to the minimum inhibitory concentrations (MICs) of EOAM (2048 µg/mL), AMO (4 µg/mL), and OXA (8 µg/mL), alone, as well as the combinations EAO (64 µg/mL of EOAM and 0.12 µg/mL AMO), ½ EAO (32 µg/mL EOAM and 0.06 µg/mL AMO), EOX (128 µg/mL EOAM and 0.12 µg/mL OXA), and ½ EOX (64 µg/mL EOAM and 0.06 µg/mL OXA).

After 24 h, all the treatments were able to reduce the melanization of the larvae and increase the viability of the hemocytes in the hemolymph (Figure 6). EOA and ½ EOA (Figure 6A) did not differ from the PBS control (white), in which the synergistic concentration reduced melanization by 61.29%. The two treatments differed in the number of hemocytes (*p* = 0.0324), with an increase in viability of 82.22% for ½ EOA, which may indicate an immunostimulatory effect on *G. mellonella* (Figure 6B).

Analysis of the hemolymph allows the study of the immune response to infection and the interaction between the host and the pathogen. Alnezary et al. [1] reported that mean hemocyte densities were significantly higher for *N. sativa* EO and its synergistic combination with vancomycin. Although the concentration of hemocytes in the hemolymph varies throughout the insect's life, its rate increases significantly during bacterial infection, indicating an increase in the organism's immune response, which can vary depending on the infectious agent [35].

The rate of melanization in *G. mellonella* also depends on the virulence of the infecting pathogen. Treatments with the synergistic concentration of EOX and the subsynergistic concentration of ½EOX differed significantly in reducing melanization in the hemolymph (*p* = 0.0039), but showed similar effects in increasing cell viability (*p* = 0.3109). Bezerra‐Filho et al. [17] also observed a reduction in melanin production induced by *S. aureus* infection. Subinhibitory concentrations of *E. brejoensis* EO reduced this production by up to 55.41% after 3 h of infection.

According to Ménard et al. [36], the degree of melanization may be related to the virulence of the microorganism. In the case of bacteria, virulence is directly related to survival in the host. Different mechanisms can be activated to try to circumvent the immune system and ensure that the bacteria remain in the body. Therefore, the results suggest that, as well as suppressing the virulence mechanisms of MRSA, the treatments also acted by immunomodulating the immune response of *G. mellonella* larvae.

## 4 Conclusions

Significant synergistic effects were observed between EOAM and the tested antibiotics, resulting in a significant reduction in the concentrations required to inhibit bacterial growth for both compounds. At synergistic concentrations, the combinations enabled better antibiotic performance, likely due to different mechanisms of action against the MRSA strain, which can be attributed to the diversity of terpenes present in the EO composition. These properties are essential for the development of adjuvants to conventional antibiotics, where therapeutic efficacy is enhanced by the combined action of EOAM with AMO and OXA. The in vivo tests with *G. mellonella* larvae confirmed the in vitro results, showing that the treatments protected the larvae from *S. aureus* infection and significantly increased survival rates. The results provide important data for future investigation into the development of combination therapies.

## Author Contributions

**Amanda Vieira de Barros**: conceptualisation, validation, methodology, formal analysis, research, and writing – original draft. **Bruno Oliveira de Veras**: supervision, methodology, conceptualisation, writing – review and editing, and data curation. **Henrique Nelson Pereira Costa Júnior**: methodology. **Raudiney Frankilin Vasconcelos Mendes**: methodology. **Patryck Érmerson Monteiro dos Santos**: methodology and writing – review and editing. **Jael Fernandes Tavares**: methodology. **Daniela Maria do Amaral Ferraz Navarro**: methodology. **Rafael Matos Ximenes**: methodology. **Patrícia Maria Guedes Paiva**: methodology. **Clovis Macedo Bezerra Filho**: writing – review and editing. **Josinaldo Alves da Silva**: methodology**. Márcia Vanusa da Silva**: supervision. **Maria Betânia Melo de Oliveira**: supervision, conceptualisation, and writing – review and editing.

## Ethics Statement

The animal ethics for this study were approved by the Research Ethics Committee—CEP of the Federal University of Pernambuco (Approval number: 0053/20).

## Conflicts of Interest

The authors declare no conflicts of interest.

## Data Availability

The authors have nothing to report.
